# A systematic review on surgical treatment of primary epigastric hernias

**DOI:** 10.1007/s10029-019-02017-4

**Published:** 2019-08-17

**Authors:** L. Blonk, Y. A. Civil, R. Kaufmann, J. C. F. Ket, S. van der Velde

**Affiliations:** 1 Department of Surgery, Amsterdam UMC, Vrije Universiteit Amsterdam, De Boelelaan 1117, 1081 HV Amsterdam, The Netherlands; 2 Department of Surgery, Tergooi, Van Riebeeckweg 212, 1213 XZ Hilversum, The Netherlands; 3 Medical Library, Vrije Universiteit Amsterdam, De Boelelaan 1117, 1081 HV Amsterdam, The Netherlands

**Keywords:** Epigastric hernia, Primary hernia, Repair, Recurrence, Mesh

## Abstract

**Objective:**

In this systematic review, we evaluated all literature reporting on the surgical treatment of primary epigastric hernias, primarily focusing on studies comparing laparoscopic and open repair, and mesh reinforcement and suture repair.

**Methods:**

A literature search was conducted in Embase.com, PubMed and the Cochrane Library up to 24 April 2019. This review explicitly excluded literature on incisional hernias, ventral hernias not otherwise specified, and isolated (para)umbilical hernias. Primary outcome measures of interest were early and late postoperative complications.

**Results:**

We obtained a total of 8516 articles and after a strict selection only seven retrospective studies and one randomised controlled trial (RCT) on treatment of primary epigastric hernia were included. In one study (RCT) laparoscopic repair led to less postoperative pain (VAS) compared to open repair (3.6 versus 2.4, *p* < 0.001). No significant differences in early postoperative complications and recurrences were observed. Mesh reinforcement was associated with lower recurrence rates than suture repair in two studies (2.2% versus 5.6%, *p*  = 0.001 and 3.1% versus 14.7%, *p* = 0.0475). This result was not sustained in all studies. No differences were observed in early postoperative complications after mesh or suture repair.

**Conclusions:**

This review demonstrated that studies investigating surgical treatment of primary epigastric hernias are scarce. The best available evidence suggests that mesh reinforcement in primary epigastric hernia repair possibily leads to less recurrences and that laparoscopic repair leads to less postoperative pain. Due to the high risk of selection bias of included studies and heterogenic study populations, no clear recommendations can be conducted. High-quality studies with well-defined patient groups and clear endpoints, primarily focusing on primary epigastric hernias, are mandatory.

**Electronic supplementary material:**

The online version of this article (10.1007/s10029-019-02017-4) contains supplementary material, which is available to authorized users.

## Introduction

The term ventral hernia is a collective term used for both primary and incisional hernias in various parts of the abdominal wall. There are various classification systems for ventral hernias, regarding defect size, location, contamination, and previous repairs. The most well-known classification system for primary ventral hernias was outlined by the European Hernia Society (EHS) [1]. This scoring system classifies primary ventral hernias into midline hernias (epigastric and umbilical) and lateral hernias (Spigelian and lumbar). Additionally, these hernias are classified by defect size: small (< 2 cm), medium ( ≥ 2–4 cm), and large (≥ 4 cm) [1]. Unfortunately, in research this classification is not often used and, as a consequence, the morphology of hernias is defined in various ways. As a result, outcomes of ventral hernia studies are difficult to compare [2].

Due to variability in definition of hernias in literature and lack of further specification, the incidence of primary epigastric hernias is difficult to establish. It was estimated that of all ventral hernia repairs in the United States, approximately two-third were primary ventral hernias, mostly umbilical and only a small portion were epigastric hernias [3]. Ninety percent of primary ventral hernia repairs are performed for small fascia defects under 2 cm [4].

Although repair of (small) primary ventral hernias is referred to as a simple surgical procedure, the optimal treatment strategy is still a matter of debate. Surgical repair of epigastric hernias can either be done with simple suture repair or reinforcement with mesh and can be performed via a laparoscopic or open procedure. Moreover, in clinical practice the surgical approach of these hernias may be influenced by surgeons preferences, patient characteristics, and hernia characteristics, such as comorbidity, patient expectations, defect size, hernia location and reducibility [5]. Nevertheless, there is great need for evidence-based treatment strategies for these common primary epigastric hernias, as they are rarely investigated as a separate entity. To our knowledge, no systematic review of the surgical treatment of primary epigastric hernias is available to date.

The aim of this systematic review was to assess the surgical treatment of primary ventral hernias, exclusively focusing on primary epigastric hernias, in adult patients. This review explicitly excluded literature on incisional hernias, ventral hernias not otherwise specified, and isolated (para)umbilical hernias.

## Methods

### Search strategy

This review was conducted and presented according to the Preferred Reporting Items for Systematic Reviews and Meta-analyses (PRISMA) guidelines [6]. In most studies, ventral hernia types are not separately recognisable and studies solely reporting on primary epigastric hernias are scarce. Therefore, an extensive literature search was conducted searching for all studies reporting on ventral hernia. Embase.com, PubMed and the Wiley/Cochrane Library were searched from inception up to 24 April 2019 (by LB and JCFK). The following terms were used (including synonyms and closely related words) as index terms or free-text words: ‘abdominal wall hernia’ or ‘ventral hernia’ or ‘epigastric hernia’ or ‘hernioplasty’ or ‘herniorraphy’ and ‘systematic reviews’ or ‘randomised controlled trials’ or ‘cohort studies’, excluding studies on children and conference abstracts. Duplicate articles were excluded. All languages were accepted. The full search strategies for all the databases can be found in Supplementary Information 1.

### Eligibility criteria

All original cohort studies and randomised controlled trials comparing laparoscopic and open repair, and mesh reinforcement and suture repair in adult patients with primary epigastric hernias were included in this review. Only studies reporting on primary ventral hernias including a minimum of ten patients with primary epigastric hernias were eligible.

Studies that did not specify hernia type, studies solely reporting on (para)umbilical hernias, or studies including incisional hernia repairs were excluded. If duplicate study populations were identified, the most recent or complete articles were selected prevent duplication bias. Studies published in English, Dutch or German were considered eligible for this systematic review.

### Outcome measures

Primary outcome measures of interest were early and late postoperative complications, such as wound infection, seroma, postoperative pain, recurrence, and reoperation. Secondary outcome measures were operative time and length of hospital stay.

### Study selection

Study selection was performed by two reviewers (LB and YC). Title and abstract of all articles were screened according to the predefined eligibility criteria. After initial selection, the full text was obtained and studies that met the eligibility criteria were included in this review. In case of disagreement in study selection, an independent author (SV) was consulted.

### Data extraction

Two reviewers (LB and YC) performed the data extraction. The following study variables were extracted from each article: study design, year of publication, demographics of study population, number of patients included, hernia characteristics, surgical techniques and postoperative complications, including hernia recurrence.

### Quality assessment

The Methodological Index For Non-Randomized Studies (MINORS) was used to assess the methodological quality of observational studies included in this systematic review [7]. The global ideal score for comparative studies was 24. The methodological quality of randomised controlled trials (RCTs) was assessed with the Cochrane Collaboration risk of bias assessment tool [8]. Two reviewers scored the articles based on the criteria’s listed by the assessment tools (LB and YC). Any discrepancies were resolved with an independent author (SV).

## Results

After screening 8516 studies on ventral hernia repair, only eight studies were found eligible for this systematic review on the surgical treatment of primary epigastric hernia. Most studies were excluded, because ventral hernia type was not specified, primary and incisional ventral hernias were mixed or only (para)umbilical hernias were included.

**Fig. 1:**
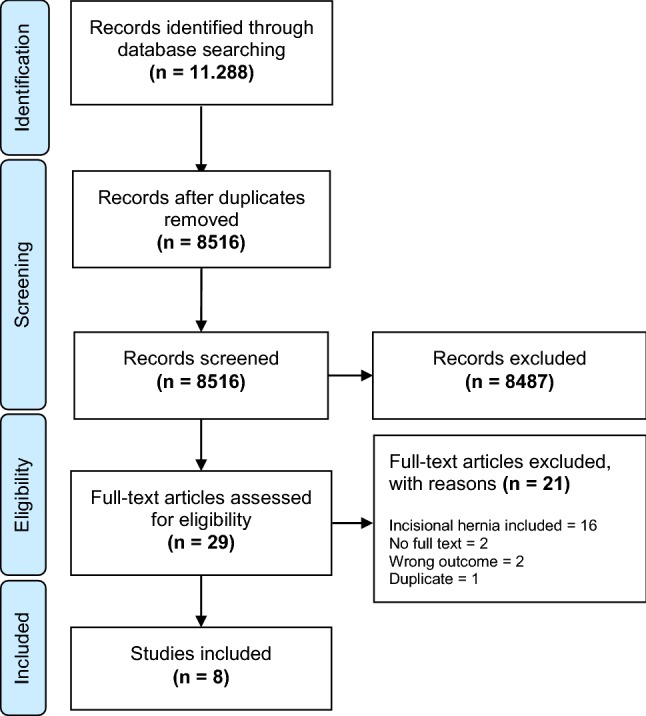
PRISMA flowchart of study selection

A total of four studies compared laparoscopic and open repair and five studies compared mesh reinforcement and suture repair. One study described both comparisons; hence, it is referred to in both sections [10]. The PRISMA flowchart of study selection is presented in Fig. 1.

### Methodological quality and risk of bias

**Fig. 2:**
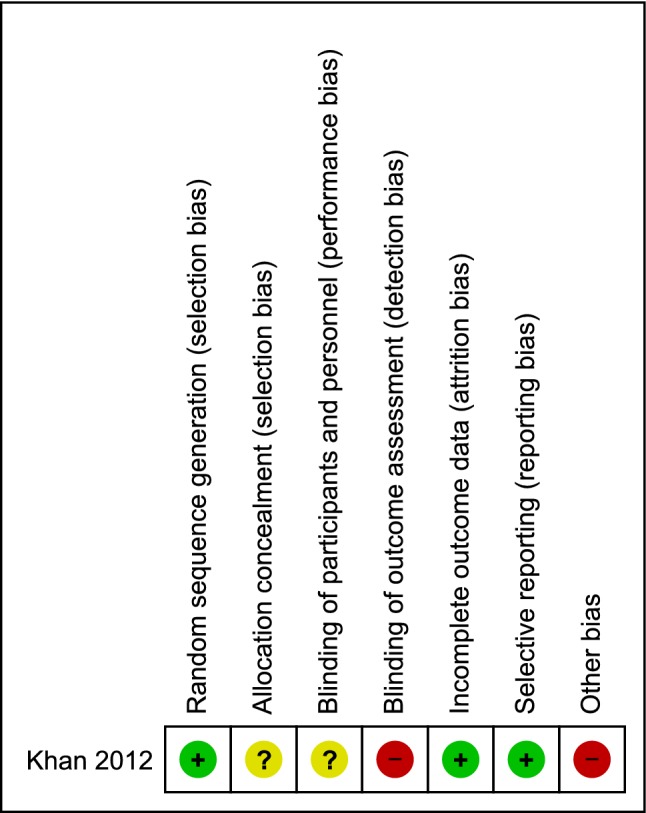
Risk of bias for randomised studies

The summary and results of methodological quality assessment of the observational studies and RCT are shown in Fig. 2 and Table 1, respectively.

**Table 1:** Risk of bias for non-randomised studies

	Clearly stated aim	Inclusion of consecutive patients	Prospective data collection	Endpoints appropriate to study aim	Unbiased assessment of study endpoint	Follow-up period appropriate to study aim	< 5% lost to follow-up	Prospective calculation of study size	Adequate control group	Contemporary groups	Baseline equivalence of groups	Adequate statistical analyses	Total
Ponten et al. [14]	2	2	0	2	0	0	1	0	2	2	1	2	16
Christoffersen et al. [13]	2	2	1	2	0	2	0	0	2	2	1	1	17
Erritzøe et al. [15]	2	2	1	2	0	2	1	0	2	2	1	2	17
Helgstrand et al. [9]	2	2	1	2	0	2	0	0	2	2	1	2	17
Bisgaard et al. [10]	2	2	1	2	0	2	0	0	2	2	1	0	16
Stabilini et al. [16]	2	2	1	2	0	2	1	0	2	2	2	2	18
Bencini et al. [12]	2	2	0	2	0	2	0	0	2	2	1	2	16

### Laparoscopic or open repair

Laparoscopic and open repair was compared in four studies [9–12]. Of these, three were retrospective cohort studies and one was a RCT, comprising a total of 2556 patients with epigastric hernias, 7819 patients with (para)umbilical hernias, and three patients with lateral hernias. None of the included studies exclusively reported on epigastric hernia. Baseline characteristics and outcome measures of the included studies are presented in Table 2.

**Table 2:** Study characteristics and outcome measures of studies comparing laparoscopic and open repair

Author	Hernia type	Surgical technique	*N*	Age (years)	Male/female ratio	BMI (kg/m^2^)	Defect size (cm)	Operative time (min)	LOS (days)	Follow-up (months)	Early postoperative complications	Late postoperative complications
Helgstrand (2013) [9] (Denmark) Retrospective cohort study	Epigastric (26%) Umbilical (74%)	Open	5601	49 (18–92)	3607/1994	NR	Median 1 (0.1–25)	NR	Median 0 (0–39)	1	NR	Readmissions: 4.4% (O)/7.7% (L) *p* < 0.001
		Laparoscopy	1182	53 (18–95) *p* < 0.001	791/391 NS		Median 3 (0.5–22) *p* < 0.001		Median 1 (0–38) *p* < 0.001			
Bisgaard et al. (2011) [10] (Denmark)	Epigastric (21%)	Open mesh or suture	3165	50 (18–92)	2098/1067	NR	NR	NR	0.4 (SD 1.9)	1	Visceral injury: 0% (O)/0.4% (L) Wound dehiscence/early recurrence: 0.3% (O)/0.4% (L)	Readmission: 5% (O)/11% (L) Reoperation: 2% (O)/3% (L)
Retrospective cohort study	Umbilical (79%)	Laparoscopy mesh	266	52 (27–90)	193/73				1.8 (SD 3.1)	1	Haematoma: 0.9% (O)/3% (L) Wound infection: 1.2% (O)/0.8% (L) Seroma: 0.4% (O)/1.9% (L)	Mortality: 0.1% (O)/0.4% (L)
Khan et al. (2012) [11] (Pakistan)	Epigastric (38%)	Open mesh	50	60 (SD 9.2)	14/36	NR	NR	48.9 ± 13.03	1.5	NR	Pain 2 h VAS: 6 (O)/4.9 (L) *p* = 0.001 Pain 24 h VAS: 3.6 (O)/2.4 (L) *p* = 0.001 Infection: NS	Recurrence: NS Mortality: 0% (O)/0% (L) NS
RCT	(Para)umbilical (62%)	Laparoscopy mesh	50	59 (SD 9.3) NS	17/33 NS			49.08 ± 11.25 NS	1.3 *p* < 0.05			
Bencini et al. (2009) [12] (Italy)	Epigastric (39%) Umbilical (56%)	Open mesh	36	Median 52 (22–81)	15/21	Median 27 (20–40)	Median 32 cm^2^ (8–140)	35 (10–145)	2 (1–11)	60 (7–80)	Overall complic.: 14% (O)/18% (L) NS Visceral injury: 0% (O)/4% (L) NS Wound infections: 8% (O)/0% (L) NS	Recurrences: 11% (O)/14% (L) NS
Retrospective cohort study	Lateral (5%)	Laparoscopy mesh	28	Median 53 (32–89) NS	12/16 NS	Median 30 (20–43) NS	Median 20 cm^2^ (8–260) NS	70 (40–165) *p* < 0.000	3 (2–10) NS	56 (1–80) NS	Seroma: 3% (O)/11% (L) N	

Means and ranges are reported unless stated otherwise

*NS* not significant, *NR* not reported, *O* open repair, *L* laparoscopic repair, *complic.* complications, *SD* standard deviation, *hrs* hours, *VAS* visual analogue scale

If no *p* value is shown, it was not reported in the concerned study

#### Early postoperative complications

Early postoperative complications were reported in all studies [9–12]. Due to the non-randomised character of data collection, Bisgaard et al. did not perform any statistical comparison between groups [10]. The RCT showed more early postoperative pain  after open repair at 2 and 24 h (*p* = 0.001) [11]. The overall complication rate, addressed in two studies, showed higher overall complication rates after laparoscopic repair, although no significant differences were reported [10, 12].

No differences in surgical site infection and seroma were reported [11, 12]. Visceral injury was solely reported after laparoscopic repair (4% and 0.4%), although no significant difference was found [10, 12].

#### Late postoperative complications

No differences in hernia recurrences were reported after laparoscopic or open repair, although only one study reported a sufficient follow-up time with a median of 60 months in the open group and 56 months in the laparoscopic group [9, 11, 12]. Readmission rates, reported in two studies, were higher after laparoscopic repair [9, 10]. Helgstrand et al. found a significant difference (7.7% versus 4.4%, *p* < 0.001) in the univariate analysis; however, after adjusting for age, hernia size, recurrent or primary hernia, and umbilical or epigastric repair, no differences were found in readmission rate between laparoscopic and open repair [9]. Readmission was mainly due to postoperative pain and wound-related complications [9, 10].

#### Operative time and length of stay

Data on length of stay and operative time were inconclusive [9–12].

### Mesh reinforcement or suture repair

A total of five retrospective studies compared open mesh reinforcement and open suture repair [10, 13–16]. Of these, one retrospective study had three treatment arms, including open onlay mesh, open intraperitoneal mesh and open suture repair [15]. Epigastric hernias were exclusively analysed in two retrospective studies, consisting of 919 patients [10, 14]. The remaining studies combined epigastric and (para)umbilical hernias [13, 15, 16]. Baseline characteristics and outcome measures of the included studies are presented in Table 3.

**Table 3:** Study characteristics and outcome measures of studies comparing mesh and suture repair

Author	Hernia type	Surgical technique	*N*	Age (years)	Male/female ratio	BMI (kg/m^2^)	Defect size (cm)	Operative time (min)	LOS (days)	Follow up (months)	Early postoperative complications	Late postoperative complications
Ponten et al. (2015) [14] (The Netherlands)	Epigastric	Open/lap Mesh	55	56 (SD 10)	32/33	28 (SD 4.8)	2.1 (SD 1.1)	46.6 (SD 17.1)	NR	NR	NR	Recurrence: 10.9% (M)/14.9% (S) NS
Retrospective cohort study		Open suture	134	49 (SD 13) *p* = .002	62/72 NS	26 (SD 4.6) *p* = .004	1.2 (SD 0.8) *p* < .001	28.6 (SD 12.7) *p* < .0001				Chronic pain: NS
Christoffersen et al. (2013) [13] (Denmark)	Epigastric (3%)	Open mesh	1348	Median 52 (18–90)	942/406	NR	1.5 (0.3–2.0)	NR	NR	21 (0–47)	Reoperation complic.: 0.2% (M)/0.1% (S) Wound bleeding:0.07% (M)/0.03% (S) Wound dehiscence: 0% (M)/0.03% (S) 30 day mortality: 0.07% (M)/0.1% (S)	Recurrence: 2.2% (M)/5.6% (S) *p* = .001
Retrospective cohort study	Umbilical (97%)	Open suture	3438	Median 47 (18–95)	2171/1267 *p* < .001		1 (0.1–2.0) *p* < .001					
Erritzøe (2013) [15] (Denmark)	Epigastric (22%) Umbilical (78%)	Open IPOM	68	Median 53 (28–82)	44/89	NR	Median 1 (0.2–8.0)	NR	NR	Median 36 (15–85)	Minor complic.: 11% (total)	Recurrence: 11.4% (total) NS Pain at rest: 6% (MI) /24% (MO) /16% (S) NS Pain during mobilisation: 10% (MI)/25% (MO)/28% (S) NS
Retrospective cohort study		Open onlay mesh	21									
		Open suture	43									
Bisgaard (2011) [10] (Denmark)	Epigastric	Open mesh	19	NR	NR	NR	NR	NR	NR	NR	Complic.: 1.4% (M)/3% (S) Readmission: 1.4% (M)/3.7% (S) Mortality: 0% (M)/ 0% (S)	Reoperation: 1.4% (M) /1.1% (S)
Retrospective cohort study		Open suture	711									
Stabilini^a^ (2009) [16] (Italy)	Epigastric (30%)	Open mesh	64	54	46/52	24.8 ± 3.1	2.8 ± 1.6	NR	1.8 (0.1–15) NS	52.9 (8–60)	Total complic.: 8% (M)/NR (S) NS	Recurrence: 3.1% (M)/14.7% (S) *p* = .0475
Retrospective cohort study	Umbilical (70%)	Open suture	34	56 NS		25.0 ± 2.2 NS	2.9 ± 2.2 NS					

Means and ranges are reported unless stated otherwise

*NS* not significant, *NR* not reported, *Lap.* Laparoscopic, *M* mesh, *S* suture, *MI* open IPOM, *MO* open onlay mesh, *complic.* Complications, *SD* standard deviation, *hrs* hours

If no *p* value is shown, it was not reported in the concerned study

^a^Emergency repair included

#### Early postoperative complications

Early postoperative complications were reported in four studies [10, 13, 15, 16]. Low complication rates were observed and there were no differences in early postoperative complications after repair with mesh reinforcement or sutures.

#### Late postoperative complications

Recurrence rates were significantly lower after mesh reinforcement in two studies, although other studies found no difference [13–16]. Of these two studies, one study of 4786 patients with a mean follow-up of 21 months showed less reoperations for recurrences after mesh reinforcement compared to suture repair (2.2 versus 5.6%, *p* = 0.001) [13]. The second study of 98 patients with a follow-up of 53 months, which also included emergency repairs, showed a recurrence rate of 3.1% after mesh repair, which was significantly lower than the recurrence rate of 14.7% after suture repair (*p* = 0.0475) [16]. The occurrence of chronic pain did not differ between suture repair or repair with mesh reinforcement [14, 15]. Patients with recurrences reported significantly more pain [14, 15].

#### Operative time and length of stay

The mean operative time, reported in one study, was longer in mesh repair compared to suture repair (47 versus 29 min, *p* < 0.0001) [14]. No differences in length of stay were reported [16].

## Discussion

This systematic review demonstrated that there are very limited studies investigating the surgical treatment of primary epigastric hernias, and the studies available are of low methodological quality. Most studies were retrospective, combined primary epigastric hernias with (para)umbilical hernias, had no long-term follow-up, and did not report on hernia characteristics, such as defect size or number of defects. Only two studies were identified that solely focussed on primary epigastric hernias.

Nevertheless, several conclusions can be conducted from this systematic review. Laparoscopic repair of primary epigastric hernia was associated with less postoperative pain. A benefit of laparoscopic repair in case of recurrence, surgical site infection, and length of stay could not be concluded from these studies. Mesh repair was associated with less recurrences compared to suture repair in two studies and no differences in early postoperative complications or chronic pain were observed.

Our study is the first systematic review to evaluate all existing literature regarding the surgical treatment of primary epigastric hernias. There have been several previous systematic reviews on laparoscopic and open repair, and mesh and suture repair including both primary and incisional hernias or solely (para)umbilical hernias [17–22].

Hajibandeh et al. investigated laparoscopic versus open repair in umbilical hernias and showed, in contrast to our results, that laparoscopic repair was associated with a lower risk of wound infection, wound dehiscence and recurrence [19]. This present study found similar recurrence rates for laparoscopic and open repair of primary epigastric hernias. This is probably attributable to an inadequate and mainly short-term follow-up in most studies. Laparoscopic repair is often associated with lower rates of surgical site infections, as incisions are small and, therefore, the risk of contamination is low. This study could not confirm a lower rate of surgical site infections after laparoscopic repair in patients with primary epigastric hernias. Extensive dissection of the abdominal wall and raising flaps for mesh fixation are important causes of postoperative pain [23]. This is confirmed by a higher rate of postoperative pain after open repair in our study.

In the past decade, four systematic reviews have been published on the use of mesh and suture in patients with primary and/or incisional ventral hernias [17, 20–22]. In accordance with our study, all reviews found lower recurrence rates after mesh repair compared to suture repair. Recurrence rates are difficult to compare across studies as different definitions of recurrence are used, such as patient-reported complaints, physical examination, or imaging of the abdominal wall. Moreover, diagnosing recurrences based on physical examination and patient-reported complaints can lead to an underestimation of recurrence rates [24]. Follow-up was often inadequate or mainly short term. Mathes et al. showed a higher risk of chronic pain for patients undergoing mesh repair, especially if the mesh was placed in sublay position [22]. In our study, no increased risk for chronic pain was observed after mesh repair [14, 15].

Studies reporting on the surgical treatment of primary epigastric hernias have several limitations. First, there is high heterogeneity in study populations, since almost all study populations consisted of both epigastric and (para)umbilical hernias, despite their different aetiologies [25]. Second, important clinical and hernia characteristics such as age, gender, BMI, and defect size were significantly different across treatment groups. This was mostly due to the retrospective character of studies available. This introduces a huge bias and should be taken into consideration when interpreting study results. Third, hernia characteristics are often not well defined and reported differently across studies. Defect size can be defined as the largest defect diameter (either width or length), hernia surface, or mesh size. Additionally, terminologies such as small, medium or large are not paired with fixed defect sizes in centimetres. Although multiple classification systems have been suggested, there is need for standardised clear definitions to report hernia characteristics [2]. Furthermore, most studies lack data about the concomitant presence of occult defects or rectus diastasis, both known to influence postoperative outcomes, especially resulting in higher rates of recurrence [26, 27].

This systematic review primarily focused on the outcomes of surgical treatment after laparoscopic and open repair, and mesh and suture repair. Therefore, several important factors, such as watchful waiting, cost efficacy, and the use of new techniques, such as robotic repair, were not addressed. Also defect closure, hernia sac resection or reduction, type and position of mesh were not investigated in this review as data of primary epigastric hernia was lacking.

In conclusion, this systematic review demonstrated that studies investigating the surgical treatment of primary epigastric hernias are scarce and of low methodological quality. Based on the available literature, no clear recommendation for surgical treatment can be made. A major concern is that primary epigastric hernias are often not well investigated as an entity and results are not separately recognisable in literature. This review stresses the need for high-quality studies solely focusing on the surgical treatment of primary epigastric hernias. Moreover, hernia and patient characteristics need to be reported in a standardised manner.

## Electronic supplementary material

Below is the link to the electronic supplementary material.

Supplementary file1 (DOCX 15 kb)
