# Supplementary material for: A systematic review on surgical treatment of primary epigastric hernias
Source: Hernia. 2019 Aug 17;23(5):847–57. doi: 10.1007/s10029-019-02017-4 (PMC6838029; doi:10.1007/s10029-019-02017-4)
Supplement: Supplementary file 1 — Supplementary file1 (DOCX 15 kb) [file 10029_2019_2017_MOESM1_ESM.docx]

**Supplementary Information 1** Literature search strategy

**Search strategy for Embase.com (24 April 2019)**

| **Search** | **Query** | **Items found** |
| --- | --- | --- |
| **#9** | #8 AND (#1 OR #2 OR #3) | **2,842** |
| **#8** | #7 NOT ('conference abstract'/it OR 'conference paper'/it OR 'conference review'/it OR 'animal experiment'/de OR 'animal model'/de OR 'case report'/de OR 'human tissue'/de OR 'nonhuman'/de) | **5,868** |
| **#7** | #6 NOT (('adolescent'/exp OR 'child'/exp OR adolescent*:ti,ab OR child*:ti,ab OR schoolchild*:ti,ab OR infant*:ti,ab OR girl*:ti,ab OR boy*:ti,ab OR teen:ti,ab OR teens:ti,ab OR teenager*:ti,ab OR youth*:ti,ab OR pediatr*:ti,ab OR paediatr*:ti,ab OR puber*:ti,ab) NOT ('adult'/exp OR 'aged'/exp OR 'middle aged'/exp OR adult*:ti,ab OR man:ti,ab OR men:ti,ab OR woman:ti,ab OR women:ti,ab)) | **10,618** |
| **#6** | #4 AND #5 | **11,172** |
| **#5** | 'hernioplasty'/exp OR 'herniorraphy'/exp OR hernioplast*:ti,ab,kw OR herniaplast*:ti,ab,kw OR repair*:ti,ab,kw OR herniorraph*:ti,ab,kw OR 'surgery'/exp OR 'surgeon'/exp OR surger*:ti,ab,kw OR surgical*:ti,ab,kw OR surgeon*:ti,ab,kw OR operation*:ti,ab,kw OR operative*:ti,ab,kw OR incisi*:ti,ab,kw OR extracti*:ti,ab,kw OR excisi*:ti,ab,kw OR invasive*:ti,ab,kw OR restorati*:ti,ab,kw OR laparoscop*:ti,ab,kw OR sutur*:ti,ab,kw OR reconstruct*:ti,ab,kw | **6,634,073** |
| **#4** | 'abdominal wall hernia'/de OR (((abdom* OR ventral* OR laparocel* OR exomphalos* OR epigastr*) NEAR/3 hernia*):ti,ab,kw) | **12,887** |
| **#3** | random* OR factorial* OR crossover* OR (cross NEXT/1 over*) OR placebo* OR (doubl* AND blind*) OR (singl* AND blind*) OR assign* OR allocat* OR volunteer* OR 'crossover procedure'/exp OR 'double blind procedure'/exp OR 'randomized controlled trial'/exp OR 'single blind procedure'/exp | **2,390,341** |
| **#2** | ('meta analysis'/exp OR 'systematic review'/exp OR ((meta NEAR/3 analy*):ab,ti) OR metaanaly*:ab,ti OR review*:ti OR overview*:ti OR ((synthes* NEAR/3 (literature* OR research* OR studies OR data)):ab,ti) OR (pooled AND analys*:ab,ti) OR (((data NEAR/2 pool*):ab,ti) AND studies:ab,ti) OR medline:ab,ti OR medlars:ab,ti OR embase:ab,ti OR cinahl:ab,ti OR scisearch:ab,ti OR psychinfo:ab,ti OR psycinfo:ab,ti OR psychlit:ab,ti OR psyclit:ab,ti OR cinhal:ab,ti OR cancerlit:ab,ti OR cochrane:ab,ti OR bids:ab,ti OR pubmed:ab,ti OR ovid:ab,ti OR (((hand OR manual OR database* OR computer*) NEAR/2 search*):ab,ti) OR ((electronic NEAR/2 (database* OR 'data base' OR 'data bases')):ab,ti) OR bibliograph*:ab OR 'relevant journals':ab OR (((review* OR overview*) NEAR/10 (systematic* OR methodologic* OR quantitativ* OR research* OR literature* OR studies OR trial* OR effective*)):ab)) NOT ((((retrospective* OR record* OR case* OR patient*) NEAR/2 review*):ab,ti) OR (((patient* OR review*) NEAR/2 chart*):ab,ti)) NOT ('editorial'/exp OR 'erratum'/de OR 'letter'/exp) | **1,177,597** |
| **#1** | cohort:ti,ab,kw OR (case:ti,ab,kw AND (control:ti,ab,kw OR controll*:ti,ab,kw OR comparison:ti,ab,kw OR referent:ti,ab,kw)) OR risk:ti,ab,kw OR causation:ti,ab,kw OR causal:ti,ab,kw OR 'odds ratio':ti,ab,kw OR etiol*:ti,ab,kw OR aetiol*:ti,ab,kw OR 'natural history':ti,ab,kw OR predict*:ti,ab,kw OR prognos*:ti,ab,kw OR outcome:ti,ab,kw OR course:ti,ab,kw OR retrospect*:ti,ab,kw OR 'epidemiology'/de | **7,391,155** |

**Search strategy for PubMed (24 April 2019)**

| **Search** | **Query** | **Items found** |
| --- | --- | --- |
| **#9** | #5 AND (#6 OR #7 OR #8) | **7,178** |
| **#8** | (((review*[tiab] OR search*[tiab] OR survey*[tiab] OR handsearch*[tiab] OR hand-search*[tiab]) AND (databa*[tiab] OR data-ba*[tiab] OR bibliograph*[tiab] OR electronic*[tiab] OR medline*[tiab] OR pubmed*[tiab] OR embase*[tiab] OR Cochrane[tiab] OR cinahl[tiab] OR psycinfo[tiab] OR psychinfo[tiab] OR cinhal[tiab] OR "web of science"[tiab] OR "web of knowledge"[tiab] OR ebsco[tiab] OR ovid[tiab] OR mrct[tiab] OR metaregist*[tiab] OR meta-regist*[tiab] OR ((predetermined[tiab] OR pre-determined[tiab]) AND criteri*[tiab]) OR apprais*[tiab] OR inclusion criteri*[tiab] OR exclusion criteri*[tiab])) OR (review[pt] AND systemat*[tiab]) OR "systematic review"[tiab] OR "systematic literature"[tiab] OR "integrative review"[tiab] OR "integrative literature"[tiab] OR "evidence-based review"[tiab] OR "evidence-based overview"[tiab] OR "evidence-based literature"[tiab] OR "evidence-based survey"[tiab] OR "literature search"[tiab] OR ((systemat*[ti] OR evidence-based[ti]) AND (review*[ti] OR literature[ti] OR overview[ti] OR survey[ti])) OR "data synthesis"[tiab] OR "evidence synthesis"[tiab] OR "data extraction"[tiab] OR "data source"[tiab] OR "data sources"[tiab] OR "study selection"[tiab] OR "methodological quality"[tiab] OR "methodologic quality"[tiab] OR cochrane database syst rev[ta] OR meta-analy*[tiab] OR metaanaly*[tiab] OR metanaly*[tiab] OR meta-analysis[pt] OR meta-synthesis[tiab] OR metasynthesis[tiab] OR meta-study[tiab] OR metastudy[tiab] OR metaethnograph*[tiab] OR meta-ethnograph*[tiab] OR Technology Assessment, Biomedical[mh] OR hta[tiab] OR health technol assess [ta] OR evid rep technol assess summ[ta] OR health technology assessment[tiab]) OR ((review*[ot] OR search*[ot] OR survey*[ot] OR handsearch*[ot] OR hand-search*[ot]) AND (databa*[ot] OR data-ba*[ot] OR bibliograph*[ot] OR electronic*[ot] OR medline*[ot] OR pubmed*[ot] OR embase*[ot] OR cochrane[ot] OR cinahl[ot] OR psycinfo[ot] OR psychinfo[ot] OR cinhal[ot] OR "web of science"[ot] OR "web of knowledge"[ot] OR ebsco[ot] OR ovid[ot] OR mrct[ot] OR metaregist*[ot] OR meta-regist*[ot] OR ((predetermined[ot] OR pre-determined[ot]) AND criteri*[ot]) OR apprais*[ot] OR inclusion criteri*[ot] OR exclusion criteri*[ot]) OR (review[pt] AND systemat*[ot]) OR "systematic review"[ot] OR "systematic literature"[ot] OR "integrative review"[ot] OR "integrative literature"[ot] OR "evidence-based review"[ot] OR "evidence-based overview"[ot] OR "evidence-based literature"[ot] OR "evidence-based survey"[ot] OR "literature search"[ot] OR ((systemat*[ti] OR evidence-based[ti]) AND (review*[ti] OR literature[ti] OR overview[ti] OR survey[ti])) OR "data synthesis"[ot] OR "evidence synthesis"[ot] OR "data extraction"[ot] OR "data source"[ot] OR "data sources"[ot] OR "study selection"[ot] OR "methodological quality"[ot] OR "methodologic quality"[ot] OR meta-analy*[ot] OR metaanaly*[ot] OR metanaly*[ot] OR meta-analysis[pt] OR meta-synthesis[ot] OR metasynthesis[ot] OR meta-study[ot] OR metastudy[ot] OR metaethnograph*[ot] OR meta-ethnograph*[ot] OR hta[ot] OR health technology assessment[ot])) | **526,453** |
| **#7** | randomized controlled trial[pt] OR controlled clinical trial[pt] OR random*[tiab] OR placebo[tiab] OR randomly[tiab] OR trial[tiab] OR groups[tiab] | **3,033,791** |
| **#6** | "Epidemiologic Studies"[Mesh] OR cohort[tiab] OR (case[tiab] AND (control[tiab] OR controll*[tiab] OR comparison[tiab] OR referent[tiab])) OR risk[tiab] OR causation[tiab] OR causal[tiab] OR "odds ratio"[tiab] OR etiol*[tiab] OR aetiol*[tiab] OR "natural history"[tiab] OR predict*[tiab] OR prognos*[tiab] OR outcome[tiab] OR course[tiab] OR retrospect*[tiab] OR follow up[tiab] OR followup[tiab] | **6,346,758** |
| **#5** | #4 NOT (animals[mh] NOT humans[mh]) | **12,807** |
| **#4** | #3 NOT (("Adolescent"[Mesh] OR "Child"[Mesh] OR "Infant"[Mesh] OR adolescen*[tiab] OR child*[tiab] OR schoolchild*[tiab] OR infant*[tiab] OR girl*[tiab] OR boy*[tiab] OR teen[tiab] OR teens[tiab] OR teenager*[tiab] OR youth*[tiab] OR pediatr*[tiab] OR paediatr*[tiab] OR puber*[tiab]) NOT ("Adult"[Mesh] OR adult*[tiab] OR man[tiab] OR men[tiab] OR woman[tiab] OR women[tiab])) | **13,645** |
| **#3** | #1 AND #2 | **14,993** |
| **#2** | "Herniorrhaphy"[Mesh] OR hernioplast*[tiab] OR herniaplast*[tiab] OR repair*[tiab] OR herniorraph*[tiab] OR "surgery" [Subheading] OR "Surgical Procedures, Operative"[Mesh] OR "Surgeons"[Mesh] OR surger*[tiab] OR surgical*[tiab] OR surgeon*[tiab] OR operation*[tiab] OR operative*[tiab] OR incisi*[tiab] OR extracti*[tiab] OR excisi*[tiab] OR invasive*[tiab] OR restorati*[tiab] OR laparoscop*[tiab] OR sutur*[tiab] OR reconstruct*[tiab] | **5,240,825** |
| **#1** | "Hernia, Ventral"[Mesh:NoExp] OR ((abdom*[tiab] OR ventral*[tiab] OR laparocel*[tiab] OR exomphalos*[tiab] OR epigastr*[tiab]) AND hernia*[tiab]) | **17,225** |

**Search strategy for Wiley/Cochrane Library (24 April 2019)**

| **Search** | **Query** | **Items found** |
| --- | --- | --- |
| **#1** | ((abdom* OR ventral* OR laparocel* OR exomphalos* OR epigastr*) AND hernia*):ti,ab,kw | **1,458** |
| **#2** | (hernioplast* OR herniaplast* OR repair* OR herniorraph* OR surger* OR surgical* OR surgeon* OR operation* OR operative* OR incisi* OR extracti* OR excisi* OR invasive* OR restorati* OR laparoscop* OR sutur* OR reconstruct*):ti,ab,kw | **275,345** |
| **#3** | #1 AND #2 | **1,360** |
| **#4** | #3 NOT ((adolescen* OR child* OR schoolchild* OR infant* OR girl* OR boy* OR teen OR teens OR teenager* OR youth* OR pediatr* OR paediatr* OR puber*) NOT (adult* OR man OR men OR woman OR women)):ti,ab,kw | **1,268** |

*Number of references per database: CDSR: 26; Protocols: 3; CENTRAL: 1238; Clinical Answers: 1.*
